# LVRT capability enhancement of DFIG based wind turbine with coordination control of dynamic voltage restorer and inductive fault current limiter

**DOI:** 10.1371/journal.pone.0221410

**Published:** 2019-08-27

**Authors:** Dongyin Zhang, Hanping Xu, Li Qiao, Lei Chen

**Affiliations:** 1 State Grid Hubei Electric Power Company Limited Economic Research Institute, Wuhan, China; 2 School of Electrical Engineering and Automation, Wuhan University, Wuhan, China; Huazhong University of Science and Technology, CHINA

## Abstract

According to the coordination control of a dynamic voltage restorer (DVR) and an inductive fault current limiter (FCL), this paper proposes an efficient low-voltage ride-through (LVRT) scheme for a doubly fed induction generator (DFIG) based wind turbine. The DVR is located to the DFIG’s stator circuit for stabilizing the terminal voltage and decreasing the generator current. The inductive FCL is connected to the DFIG’s rotor circuit for suppressing the rotor overcurrent and protecting the converter. Theoretical discussions on structure, principle and scale criterion of the combined DVR-FCL are conducted, and simulation analyses of the proposed approach to handle symmetrical and asymmetrical faults are done in MATLAB/Simulink. In this study, the dynamic characteristics of the DFIG during the faults are analyzed from multiple aspects, and a detailed comparison of the proposed approach and the single action of DVR or FCL is carried out. From the simulation results, the effectiveness and superiority of the proposed approach are well demonstrated.

## 1 Introduction

In recent years, the contradiction between the increase of energy demands and the shortage of fossil fuels has been more and more serious, and to achieve sustainable socio-economic development, promoting the penetration of renewable energy (RE) in power systems has been regarded as a critical solution [1–3]. In a sense, to construct a smart energy city, the application of micro-grids can contribute to accommodating more various RE sources and decreasing their adverse effects caused by uncertainties [4]. As a representative of RE, wind energy has obtained the fastest growth, and the cumulative installed capacity of wind power generators all over the world may be more than 800 GW by 2021 [5,6].

Note that, energy quality is a significant feature to affect the stability and security of electric power systems, and it is very crucial to stabilize wind power generators under short-circuit faults. Wind turbines (WTs) should keep the grid-connected status for a certain time, and this condition depends on the severity of faults or level of voltage sags to meet specific code demands, so called as low-voltage ride-through (LVRT) operation.

As the most widely WT, doubly fed induction generator (DFIG) has obtained considerable attention, and many different measures regarding the LVRT enhancement of DFIG have been suggested. Generally, the existing methods are classified as software and hardware approaches. The software solution is regarding an improved or updated control strategy with less cost, but it is just suitable for handling moderate fault conditions [7]. The hardware solution is to apply one or more devices with cost investment, and it has a good ability to deal with serious short-circuit faults. The literature review is presented as follows.

### 1.1 Literature review

In [8], an advanced current tracking controller is applied in the rotor-side converter (RSC). Scholars discuss how to determine a proper tracking coefficient for the controller, and the results show the transient fluctuations in the RSC can be well constrained. In [9], an available (generator side converter) GSC voltage is utilized to conduct the voltage compensation, and the DFIG’s transient flux is controlled to obtain a desirable fault current limitation. In [10], a linear-quadratic regulator is implemented in the DFIG. This regulator serves as the supplementary control to prevent converter saturation. In [11], an optimal hierarchical control structure is proposed. The primary and secondary control levels are designed, and it is found that active and reactive power oscillations in the generator can be favorably mitigated. In [12,13], two improved controllers basing fuzzy logic are used in the RSC, and the key functions of the proposed controllers are to decrease the rotor current and inhibit the DC-link voltage. In [14], scholars investigate an analytical method to determine the control parameters of the DFIG subject to the capacity limit of the RSC. On the whole, the transient stability support from the software solutions towards the DFIG may be relatively moderate, and the improvements of optimizing current reference and introducing over-modulation could be appreciatively done.

In the following, the hardware solutions based on chopper circuit, voltage compensator/restorer and FCL are reviewed. In [15], the efficacy of a DC-link chopper on diminishing the DC overvoltage is validated, nevertheless it fails to assist the demagnetization of the electrical machine post-fault. In [16], scholars propose a modified DC chopper that can be inserted in a DFIG basing series or parallel connection. Although the modified structure makes certain improvements, the rotor current is still around its safety limit (2.0 pu). In [17], a minimised series voltage compensator is applied. Since the stator flux is well controlled, the generator is allowed to ride-through the grid disturbances.

In [18,19], scholars prove that a DVR is better than a crowbar circuit to handle the transient fluctuations of a DFIG. When the DVR is to solve serious voltage decline with a longer duration, it is needed to consider sufficient energy support [20]. To deeply explore the potentials of the DVR, an enhanced voltage control basing the combination of feed-forward and feedback is proposed in [21], and an improved topological structure is discussed in [22]. Using the DVR can offer flexible transient- and steady-state response for the DFIG. On the premise of meeting the DFIG’s LVRT capability, it is recommended to reduce the DVR rating for making the solution be more practical. From this perspective, introducing a device with better economic performance to decrease the DVR rating might be an appropriate option.

Regarding the application of a FCL in a DFIG, studies focus on bridge-type [23–26] and superconducting-type FCLs [27–34]. In [23], a bridge-type FCL with bypass resistor is applied in a DFIG. The research results confirm its positive effects on reducing the flux and electromagnetic torque oscillations. In [24], the efficacy comparison of a bridge-type FCL and a series dynamic braking resistor is carried out. It is illustrated that the FCL owns better suitability than the braking resistor in stabilizing a DFIG. In [25], a nonlinear control-based modified bridge-type FCL is presented. Owing to the structure improvement, the proposed FCL outperforms the conventional bridge-type FCLs to support the LVRT operation and has quicker withdrawal action. In [26], scholars propose a capacitive bridge-type FCL to increase the grid-side voltage, and a discharging resistor is configured to dissipate excess power for protecting the RSC.

In [27,28], an active-type SFCL and a flux-coupling-type SFCL are installed at the stator of a DFIG, and the two SFCLs both appear hybrid current-limiting impedance to suppress the transient fluctuations. Although an effective reduction in the stator current is realized, there is room for mitigating the rotor current. In [29–32], the contributions of the resistive SFCL in the DFIG rotor are evaluated. The stability of the RSC is strengthened, and the DC-link overvoltage is clearly alleviated. However, it is not good at enhancing the terminal voltage, and the heat accumulation in the resistive SFCL may cause a long quench recovery time. In [33,34], the scheme design and assessment of a modified flux-coupling-type SFCL for medium-scale wind plants with multiple DFIGs are studied, and the results imply that reducing the operation loss and cumulative heat of the SFCL is of significance. From this perspective, using an inductive current-limiting device is an alternate solution [35].

It is worthy to state that, a few preliminary studies on the coordination control of a fault current limiter and an energy storage device for stabilizing a DFIG have been reported [36–40]. It is revealed that the combined utilization of two devices with different functions can bring more contributions in enhancing the transient characteristics of a DFIG. In a sense, developing this kind of study and exploring a novel combination scheme with preferable potentials are of significance.

### 1.2 Contributions of this paper

In this paper, our research group proposes the coordination control of a DVR and an inductive FCL to improve a DFIG’s LVRT capability. The DVR is located to the DFIG’s stator circuit for stabilizing the terminal voltage and decreasing the generator current. The FCL is connected to the DFIG’s rotor circuit for suppressing the rotor overcurrent and protecting the converter.

For the DFIG, employing the DVR is to offer a direct voltage compensation and an indirect current limitation. As the DVR is not good at decreasing the rotor current, a considerable DVR rating should be designed to make all LVRT criteria including the stator voltage, rotor current and electromagnetic torque be satisfied under the severe fault. When the inductive FCL cooperates with the DVR to handle the LVRT issue together, the direct contribution of the FCL in lowering the rotor current can effectively remedy the performance limitation of the DVR. Thus, the application of the FCL will bring a proper reduction in the DVR rating, and meanwhile the DVR alleviates the current-limiting pressure of the FCL.

The main contributions and novelty of this paper are summarized as follows:

1. Applying a DVR and an inductive FCL into different locations of a DFIG for its LVRT capability improvement.
2. Clarifying the theoretical effects of the combined DVR-FCL on the DFIG’s transient behaviors.
3. Evaluating the effectiveness of the proposed approach in a typical DFIG under symmetrical and asymmetrical faults.
4. Performing a detailed comparative study of the proposed approach and the single action of DVR or FCL, in terms of the DFIG’s voltage-current fluctuations, power delivery stability and electromagnetic torque oscillations.

### 1.3 Organization of this paper

The arrangement of this paper is as follows. Section 2 conducts the theoretical presentation of a DFIG. In Sections 3–4, the structural principle and coordinated method of a DVR and an inductive FCL for a DFIG are elaborated. In Section 5, simulation evaluation and performance comparison are implemented. Section 6 summarizes the main findings and explores the improvements in the future.

## 2 Theoretical Presentation of a typical DFIG based WT

**Fig 1:**
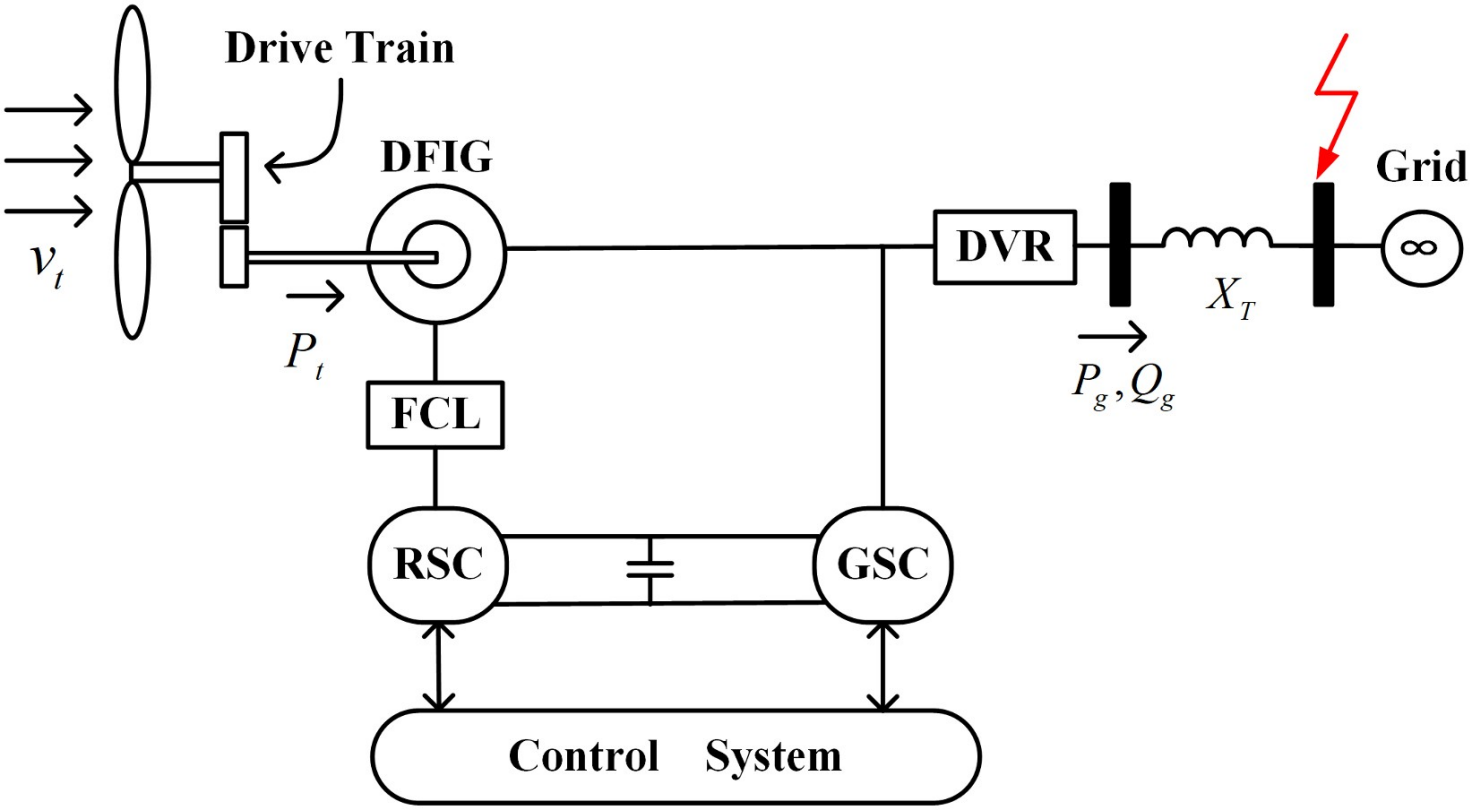
Schematic of a DFIG based WT with a DVR and an inductive FCL.

Fig 1 shows the schematic of a typical DFIG based WT, which is accessed to the main network through a power transformer. By referring to [41], the modeling equations are formulated as:

$$\vec{V_{s}}=R_{s}\vec{i_{s}}+d\vec{\psi_{s}}/dt+j\omega_{s}\vec{\psi_{s}}$$ (1)

$$\vec{V_{r}}=R_{r}\vec{i_{r}}+d\vec{\psi_{r}}/dt+j(\omega_{s}-\omega_{r})\vec{\psi_{r}}$$ (2)

$$\vec{\psi_{s}}=L_{s}\vec{i_{s}}+L_{m}\vec{i_{r}}$$ (3)

$$\vec{\psi_{r}}=L_{m}\vec{i_{s}}+L_{r}\vec{i_{r}}$$ (4)

where $\vec{i_{}},\vec{V_{}},\vec{\psi_{}}$, *R*, *L* are the current, voltage, flux, resistance as well as inductance, respectively. Subscripts *s*, *r* are the stator and rotor, respectively. It is obtained that *L*_*s*_ = *L*_*sσ*_ + *L*_*m*_ and *L*_*r*_ = *L*_*rσ*_ + *L*_*m*_, and *L*_*sσ*_/*L*_*rσ*_ is the leakage inductance.

In light of the Eqs (3) and (4), the stator and rotor currents are signified as:

$$\vec{i_{s}}=\vec{\psi_{s}}/L^{\prime }{}_{s}-k_{r}\vec{\psi_{r}}/L^{\prime }{}_{s}$$ (5)

$$\vec{i_{r}}=-k_{s}\vec{\psi_{s}}/L^{\prime }{}_{r}+\vec{\psi_{r}}/L^{\prime }{}_{r}$$ (6)

where $L^{\prime }{}_{s}=L_{s}-L^{2}{}_{m}/L_{r}$ and $L^{\prime }{}_{r}=L_{r}-L^{2}{}_{m}/L_{s}$ are deduced; *k*_*s*_ and *k*_*r*_ are expressed as *k*_*s*_ = *L*_*m*_ / *L*_*s*_ and *k*_*r*_ = *L*_*m*_ / *L*_*r*_, respectively.

**Fig 2:**
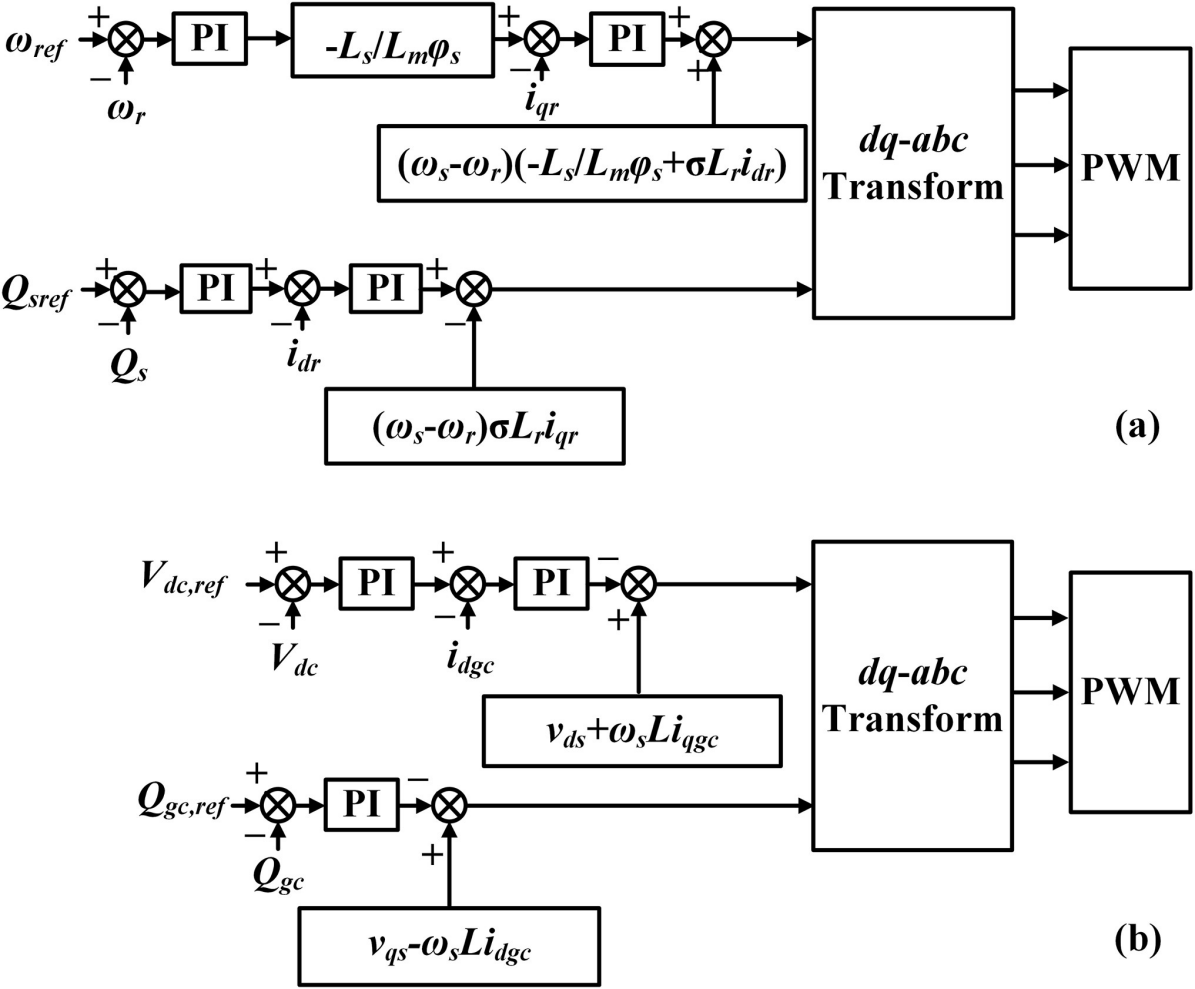
Control block diagram of the DFIG converters. (a) RSC, (b) GSC.

Fig 2 shows the control block diagram of the DFIG converters. For the DFIG, the RSC is to adjust the rotor current and reactive power, and the GSC is to regulate the DC-link voltage and grid-side current [42,43]. A brief description of the adopted control strategy is as follows: (1) The rotor angular frequency *ω*_*r*_ is measured from the wind conditions, and the reference *ω*_*ref*_ is concerning maximum power point tracking. In light of the deviation *Δω*, the rotor current reference *i*_*qr-ref*_ can be gained, and then a classical proportional-integral controller is used for the rotor current regulation. (2) The reference *V*_*dc*,*ref*_ is regarding the DC-link nominal voltage, and a comparing control loop is constructed for the DC-link voltage maintenance. (3) The reactive power adjustments are implemented by both the RSC with the reference *Q*_*sref*_ and the GSC with the reference *Q*_*gc*,*ref*_ [44]. Since this study focuses on exploring a hardware solution based on the combined DVR-FCL for the DFIG, our research group does not conduct additional modifications on the theory and control strategy of the DFIG controllers.

## 3 Configuration of the DVR and its effects on the DFIG

**Fig 3:**
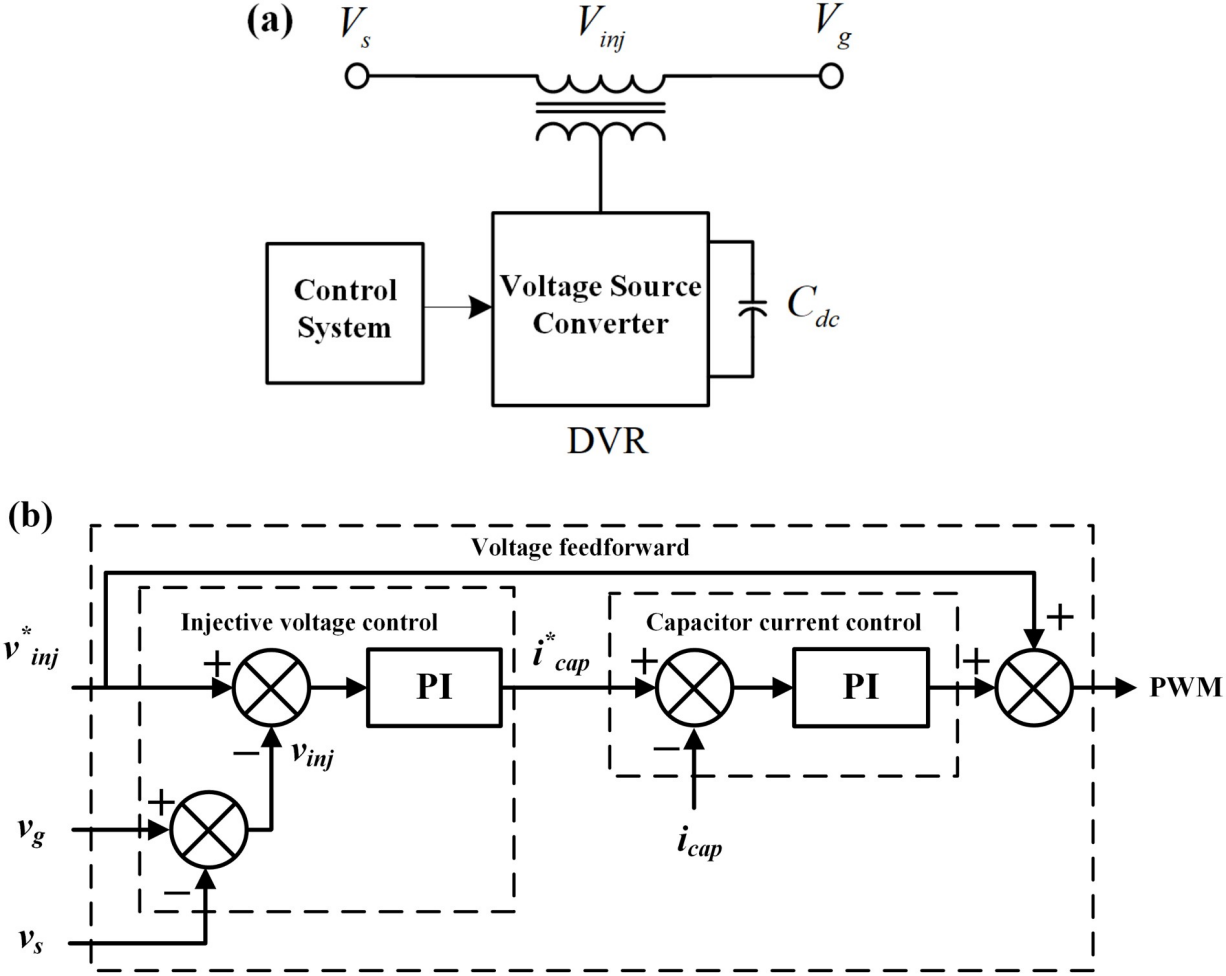
Description of the DVR. (a) Schematic, (b) control block diagram.

Fig 3a denotes the schematic of a DVR, and Fig 3b gives the control block diagram [45,46]. From the suggested control, the DVR may inject a highly flexible and adjustable voltage in series with the generator terminal.

In this study, a voltage-drop coefficient *A*_1_ (0≤*A*_1_≤1) is introduced for the DFIG, and the terminal voltage is expressed as:

$$\vec{V_{s}}=\{\begin{array}{ll} V_{s}e^{j\omega_{s}t} & t<t_{0}\\ (1-A_{1})V_{s}e^{j\omega_{s}t} & t\geq t_{0} \end{array}$$ (7)

where *t*_0_ is the fault occurrence time.

Considering the voltage compensation by the DVR, its function is represented by a voltage-increase coefficient *A*_*DVR*_ (0≤*A*_*DVR*_≤*A*_1_). Thereupon, the DFIG’s terminal voltage will be rewritten as:

$$\vec{V_{s}}=\{\begin{array}{ll} V_{s}e^{j\omega_{s}t} & t<t_{0}\\ (1-A_{1}+A_{DVR})V_{s}e^{j\omega_{s}t} & t\geq t_{0} \end{array}$$ (8)

By referring to the constant-linkage theory [47], the stator flux is:

$$\vec{\psi_{s}}=\{\begin{array}{ll} \frac{L_{s}V_{s}}{R_{s}+j\omega_{s}L_{s}}e^{j\omega_{s}t}+\frac{R_{s}L_{s}I_{r}}{R_{s}+j\omega_{s}L_{s}}e^{j\omega_{s}t} & t<t_{0}\\ \frac{L_{s}(1-A_{1}+A_{DVR})V_{s}}{R_{s}+j\omega_{s}L_{s}}e^{j\omega_{s}t}+\frac{R_{s}L_{s}I_{r}}{R_{s}+j\omega_{s}L_{s}}e^{j\omega_{s}t}+\vec{\psi_{s0}}e^{-R_{s}t/L_{s}} & t\geq t_{0} \end{array}$$ (9)

where $\vec{\psi_{s0}}$ is to describe the natural component of the stator flux, and its expression is:

$$\vec{\psi_{s0}}=\frac{L_{s}(A_{1}-A_{DVR})V_{s}e^{j\omega_{s}t_{0}}}{R_{s}+j\omega_{s}L_{s}}$$ (10)

By ignoring *R*_*s*_ in the Eq (9), the stator flux can be rewritten as:

$$\vec{\psi_{s}}=\{\begin{array}{ll} \frac{V_{s}}{j\omega_{s}}e^{j\omega_{s}t} & {t<t}_{0}\\ \frac{(1-A_{1}+A_{DVR})V_{s}}{j\omega_{s}}e^{j\omega_{s}t}+\frac{(A_{1}-A_{DVR})V_{s}e^{j\omega_{s}t_{0}}}{j\omega_{s}}e^{-\frac{R_{s}}{L_{s}}t} & t\geq t_{0} \end{array}$$ (11)

Thus, in combination with the Eq (3), the stator current will be deduced as:

$$\vec{i_{s}}=\frac{(A_{1}-A_{DVR})V_{s}e^{j\omega_{s}t_{0}}}{j\omega_{s}L_{\text{s}}}e^{-\frac{R_{s}}{L_{s}}t}\text{+}\frac{(1-A_{1}+A_{DVR})V_{s}}{j\omega_{s}L_{\text{s}}}e^{j\omega_{s}t}-\frac{L_{m}\vec{i_{r}}}{L_{s}}$$ (12)

From the above theoretical derivations, introducing the DVR is able to offer a direct effect on alleviating the stator-voltage drop, and meanwhile, the fault current in the stator side can be potentially suppressed.

## 4 Structure, control and influence of the inductive FCL for the DFIG

**Fig 4:**
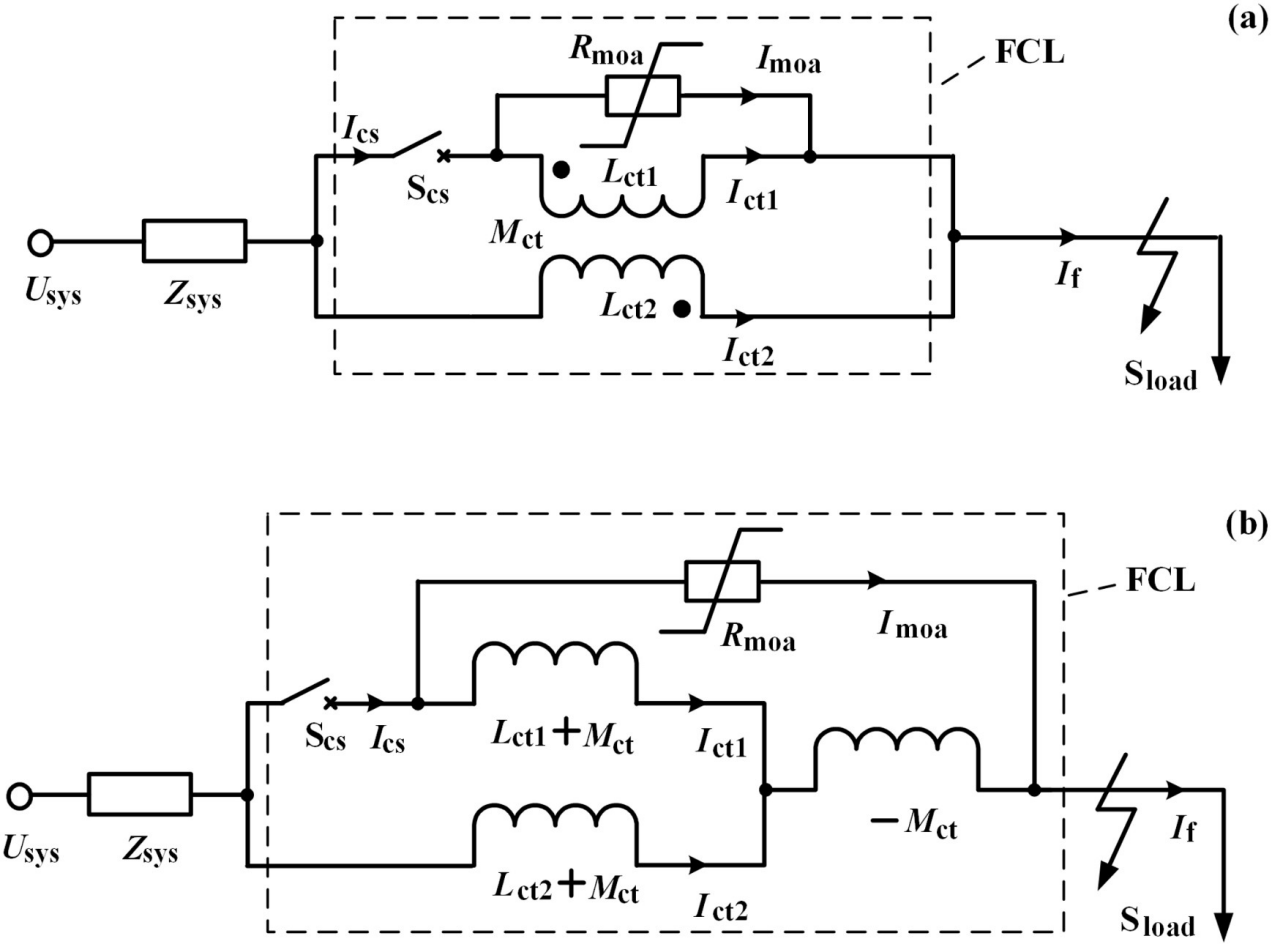
Topological structure of the inductive FCL. (a) Main connection, (b) equivalent circuit.

After the expected effects of the DVR are exploited, the inductive FCL should be quickly triggered. Fig 4 shows the topological structure of the inductive FCL, and by controlling the status of the switch S_cs_, the current-limiting inductance of *Z*_*FCL*_ ≈ j*ωL*_*ct2*_ can be activated in time [48,49].

**Fig 5:**
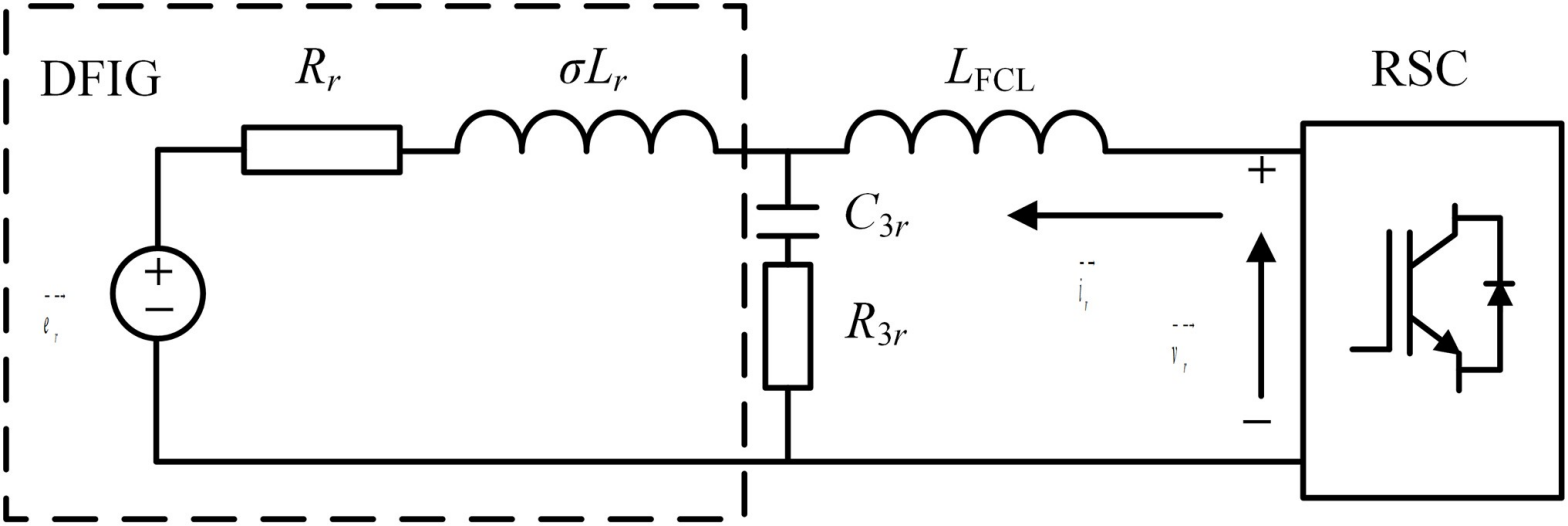
Equivalent circuit analysis of the RSC with the FCL.

Fig 5 shows the equivalent circuit of the RSC with the inductive FCL. Herein, *R*_3*r*_ and *C*_*3r*_ represent the filtering resistance and capacitance, respectively. This resistance-capacitance branch can join the leakage inductance of the FCL and the rotor to form a LCL filter with the low-pass feature. Then, the rotor voltage mainly appears the frequency component, and according to the rotor back-electromagnetic force (EMF) [50], the voltage equation is derived as:

$$\vec{V_{r}}=R_{r}\vec{i_{r}}+(\sigma L_{r}+L_{FCL})\frac{d}{dt}\vec{i_{r}}+\vec{e_{r}}$$ (13)

where *σL*_*r*_ is denoted as the rotor transient inductance; $\vec{e_{r}}$ is the EMF induced at the rotor side, and its expression is:

$$\vec{e_{r}}=\{\begin{array}{ll} k_{s}V_{s}e^{j\omega_{s}t} & {t<t}_{0}\\ k_{s}s(1-A_{1}+A_{DVR})V_{s}e^{j\omega_{s}t}-(1-s)k_{s}(A_{1}-A_{DVR})V_{s}e^{-j(1-s)\omega_{s}t}e^{-\frac{R_{s}}{L_{s}}t} & t\geq t_{0} \end{array}$$ (14)

where *s* is used to denote the slip with the range of [-0.3, 0.3].

During the transient process, the rotor current could be contributed by three components [51]. The first component is feed by the voltage applied to the rotor circuit; the second component is feed by the forced component of the rotor open voltage; the third component is a dc component damping in an exponential way. Thus, considering the impacts of the combined DVR-FCL, the rotor current is expressed as:

$$\begin{array}{c} \vec{i_{r}}=\frac{V_{rc}-k_{s}s(1-A_{1}+A_{DVR})V_{s}}{R_{r}+js\omega_{s}(\sigma L_{r}+L_{FCL})}e^{js\omega_{s}t}\text{+}\frac{k_{s}(1-s)(A_{1}-A_{DVR})V_{s}}{R_{r}-j(1-s)\omega_{s}(\sigma L_{r}+L_{FCL})}e^{-j(1-s)\omega_{s}t}e^{-\frac{R_{s}}{L_{s}}t}\\ +\left[\frac{V_{r}-V_{rc}-k_{s}s(A_{1}-A_{DVR})V_{s}}{R_{r}+js\omega_{s}(\sigma L_{r}+L_{FCL})}-\frac{k_{s}(1-s)(A_{1}-A_{DVR})V_{s}}{R_{r}-j(1-s)\omega_{s}(\sigma L_{r}+L_{FCL})}\right]e^{-\frac{R_{r}}{L_{r}+L_{FCL}}t} \end{array}$$ (15)

where *V*_*rc*_ denotes the rotor open voltage.

From the Eq (15), adjusting the parameters of the DVR (*A*_*DVR*_) and the FCL (*L*_*FCL*_) will both affect the rotor current limitation. Herein, a simplified calculation of the rotor current is done, and it is assumed that *A*_1_ = 1, *k*_*s*_ ≈ 1, *A*_*DVR*_ = 0 and *R*_*r*_ ≈ 0. Thereupon, the maximum rotor current is approximatively written as:

$${\left|\vec{i_{r}}\right|}_{\text{max}}\leq \frac{V_{r}+sV_{s}}{s\omega_{s}(\sigma L_{r}+L_{FCL})}$$ (16)

According to the Eq (16), the relation between the maximum rotor current and the inductance *L*_*FCL*_ can be roughly determined, and after the DVR is applied, the parameter setting of *L*_*FCL*_ will be properly decreased.

## 5 Simulation analysis

To validate the effectiveness and feasibility of the proposed approach, simulation analyses are done in MATLAB, and Table 1 summarizes the parameters. Regarding the design criteria for DVR and FCL, an explanation is given. For only using a DVR in a DFIG, the DVR capacity can be equal to the rated power of the DFIG, and this original design criterion is adopted in [21]. Since the application of the FCL can alleviate the DVR rating, this study doesn't use the original design criteria. Correspondingly, it is designed that the DVR capacity is half of the DFIG power rating, and the FCL rating is based on a reasonable reduction in the flux-coupling-type FCL rating [27]. As the inductive FCL has a relatively simple structure, the economic performance of the inductive FCL is generally superior to that of the DVR [52], and it means that the proposed scheme (a half compensation DVR and an inductive FCL) may offer better economic performance than a full compensation DVR. Note that, the parameters of the FCL and DVR have not been fully optimized. Aiming at their different costs and contributions to the DFIG’s LVRT operation, a detailed capacity optimization study will be presented in another report.

**Table 1:** Main parameters of the DFIG with the combined DVR-FCL.

FCL	Primary/Secondary/Mutual inductance	6 mH/6 mH/5.99 mH
	Coupling Coefficient *k*	0.999
DVR	Rated capacity	0.75 MW
	Filtering capacitance	0.1 mH
	Filtering inductance	1 μF
	Switching frequency	10 kHz
	Series transformer ratio	1
DFIG based WT	Rated capacity	1.5 MW
	Rated wind speed	11 m/s
	Stator voltage / frequency	690 V / 50 Hz
	Rotor voltage / frequency	2370 V / 12 Hz
	Stator resistance / leakage inductance	0.023 pu / 0.18 pu
	Rotor resistance / leakage inductance	0.016 pu / 0.16 pu
	DC-link voltage	1150 V

### 5.1 Study of the symmetrical fault

**Fig 6:**
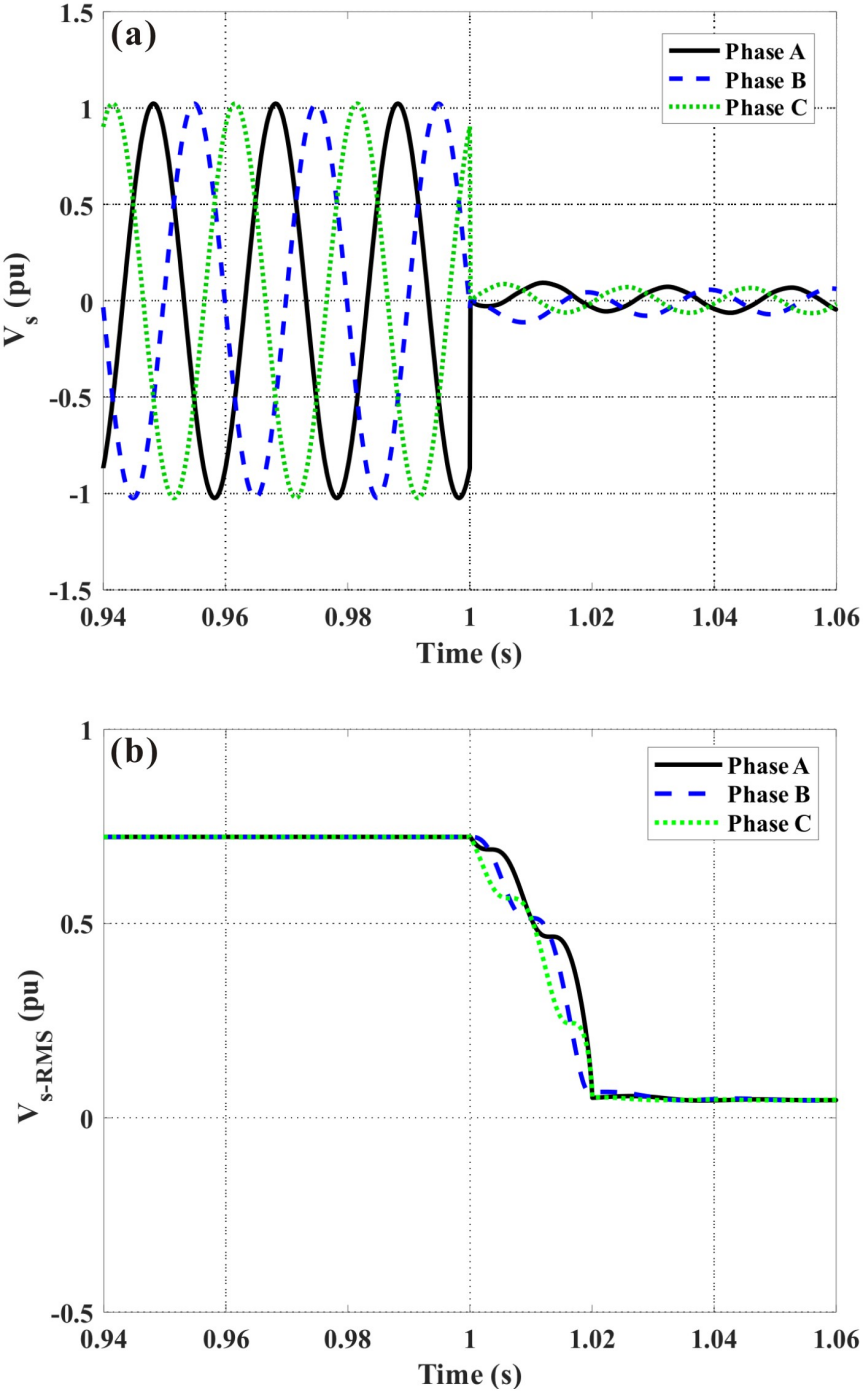
DFIG terminal voltage subject to the symmetrical fault. (a) Instantaneous value, (b) RMS value.

It is simulated that a three-phase fault occurs at *t* = 1 s, and the fault duration is 200 ms. Fig 6 shows the DFIG terminal voltage in the event of without auxiliary. Herein, the range of time-axis is set as [0.94 s, 1.06 s], and it is expected to more clearly show the voltage change before and after the fault. Both the instantaneous value and Root-Mean-Square (RMS) value are given, and when the steady peak value of the terminal voltage (standard sinusoidal wave) is 1.0 pu, the RMS value is calculated as $\frac{1}{\sqrt{2}}=0.707\text{pu}$. Under the fault, the generator voltage declines sharply, and the caused transient phenomena are very strong. During the simulations, we consider four different cases, which are expressed as without auxiliary, only with the DVR, only with the FCL, and with the DVR-FCL. The criteria regarding the LVRT behaviors are described as that: (1) The generator voltage satisfies the Denmark code. The DFIG needs to hold its connection state at least for 150 ms when the terminal voltage drops to 20% of the nominal level. Meanwhile, the generator voltage should recovery to 75% of nominal level within about 0.7 s. (2) The limit of the rotor current is set as 2.0 pu [53,54]. (3) The DC-link voltage is lower than 1.35 kV. (4) The electromagnetic torque is not exceeding 2 ~ 2.5 pu [55–57].

**Fig 7:**
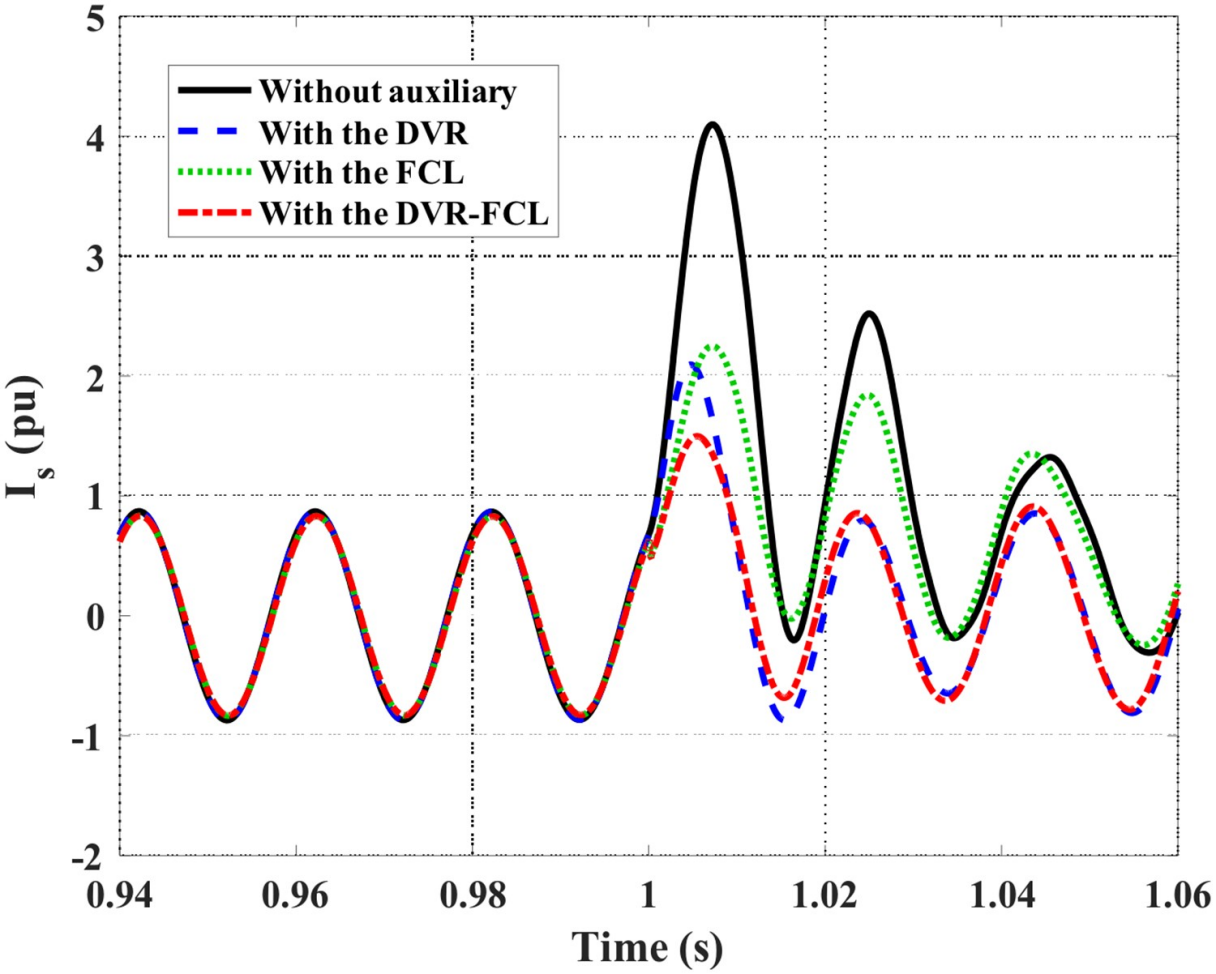
Stator current of the DFIG subject to the symmetrical fault.

**Fig 8:**
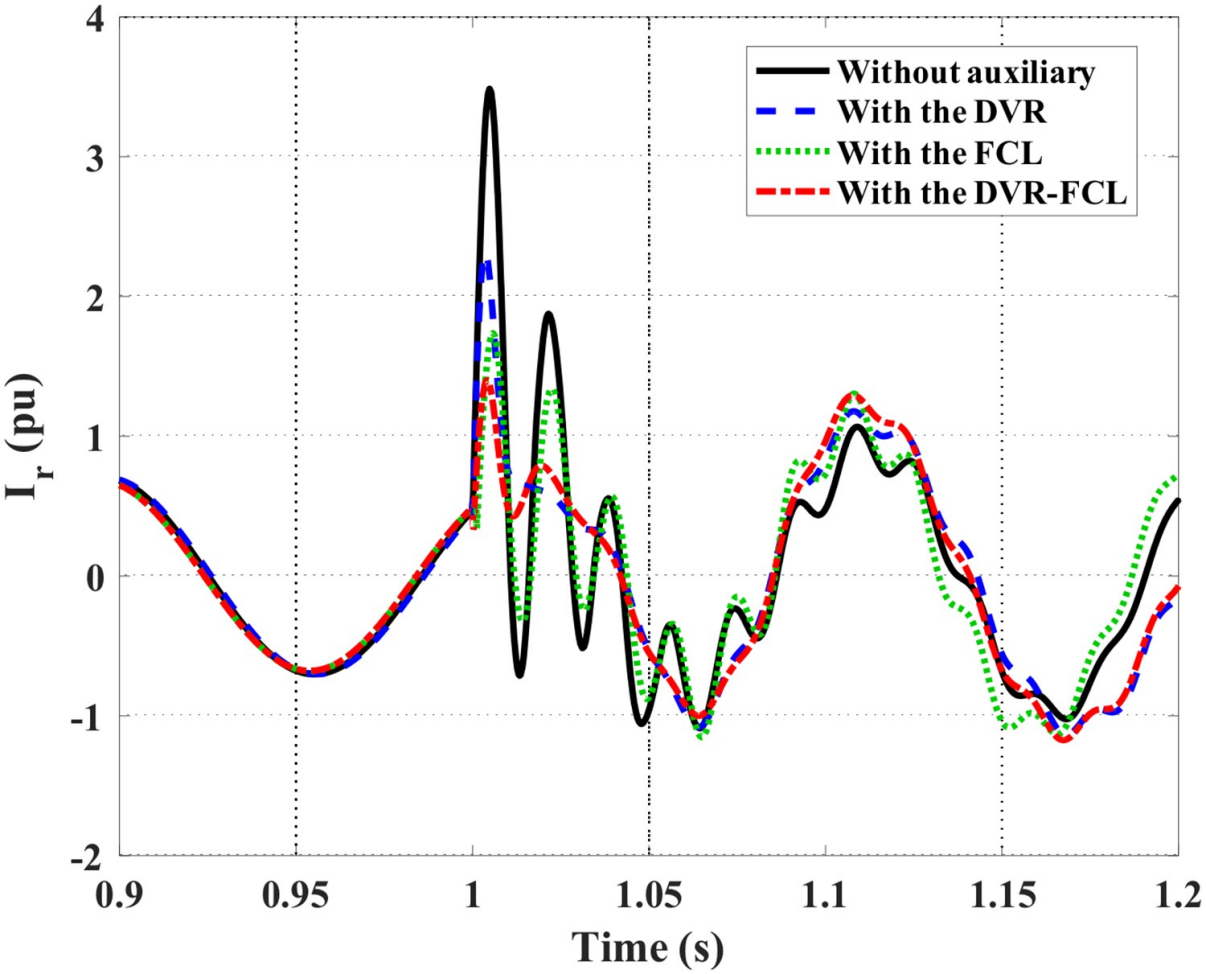
Rotor current of the DFIG subject to the symmetrical fault.

Figs 7 and 8 show the DFIG stator and rotor current characteristics. Obviously, the combined DVR-FCL performs the best functions in suppressing the fault currents, and an adequate safety margin will be caused for supporting the LVRT operation. It should be pointed out that, only with the DVR is insufficient to make the DFIG ride through the fault, as the rotor current is still larger than the allowable limit.

**Fig 9:**
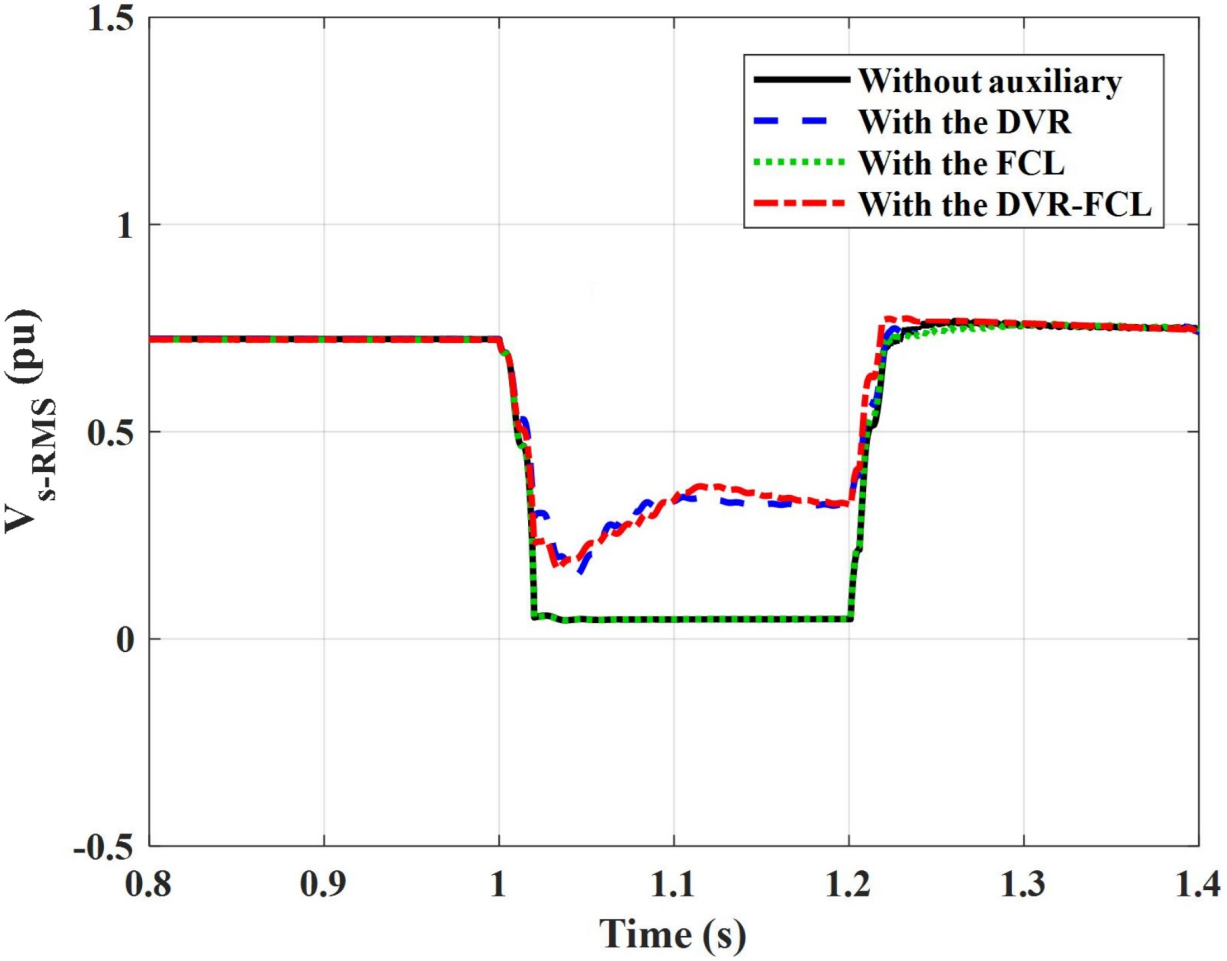
RMS value of the DFIG terminal voltage subject to the symmetrical fault.

Fig 9 denotes the RMS feature of the DFIG terminal voltage, where the phase-A is selected. The symmetrical fault causes a very serious voltage decline, and using the DVR can compensate the voltage to 50% of the nominal level. Whereas, the FCL has almost no influence on the terminal voltage. The simulation results show that the terminal voltage will start recovery at 1.2 s, where the short-circuit fault is exactly removed. According to the Denmark code, it implies that, if the wind generator expects to ride through a short-circuit fault with a little longer duration (200 ms), a higher terminal voltage level during the fault should be reached (25% of the nominal level). From this perspective, considering the favorable voltage compensation by the DVR-FCL, the DFIG can be soundly connected to the main network for 0.46 s, which is greatly more than the fault duration. Therefore, an adequate time margin is obtained for keeping the connection state of the DFIG.

In practice, when the DFIG fails to meet the LVRT criteria, it will be disconnected, and the terminal voltage cannot be recovered timely until the reconnection is done. To observe the possible dynamic fluctuations, we do not simulate the action of shutting down the wind generator even if it has an insufficient LVRT capability. Thus, for without auxiliary, the DFIG terminal voltage can favorably recover to the normal condition after the fault is removed. Also, the combined DVR-FCL has a moderate ability to accelerate the voltage recovery.

**Fig 10:**
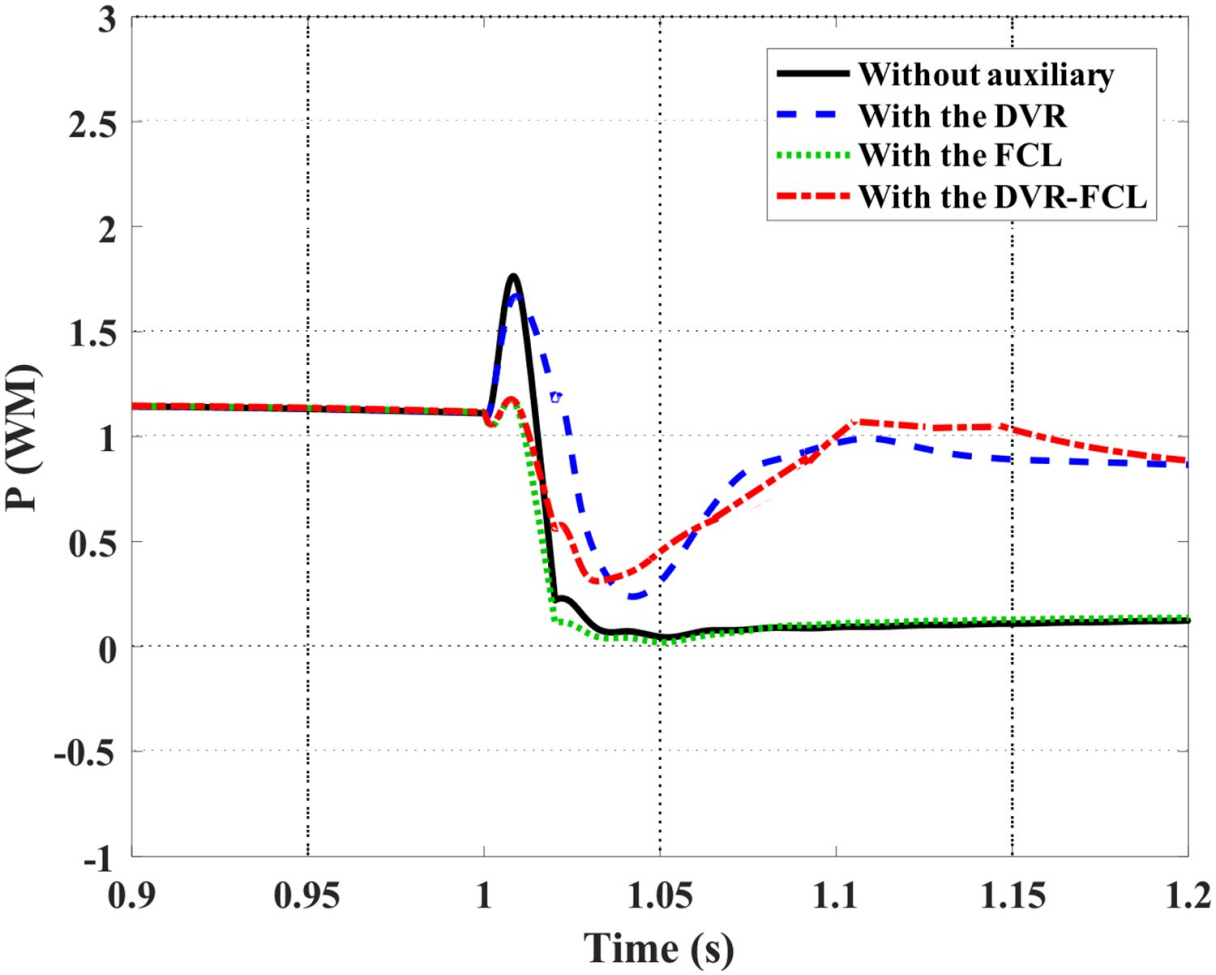
Active power of the DFIG subject to the symmetrical fault.

**Fig 11:**
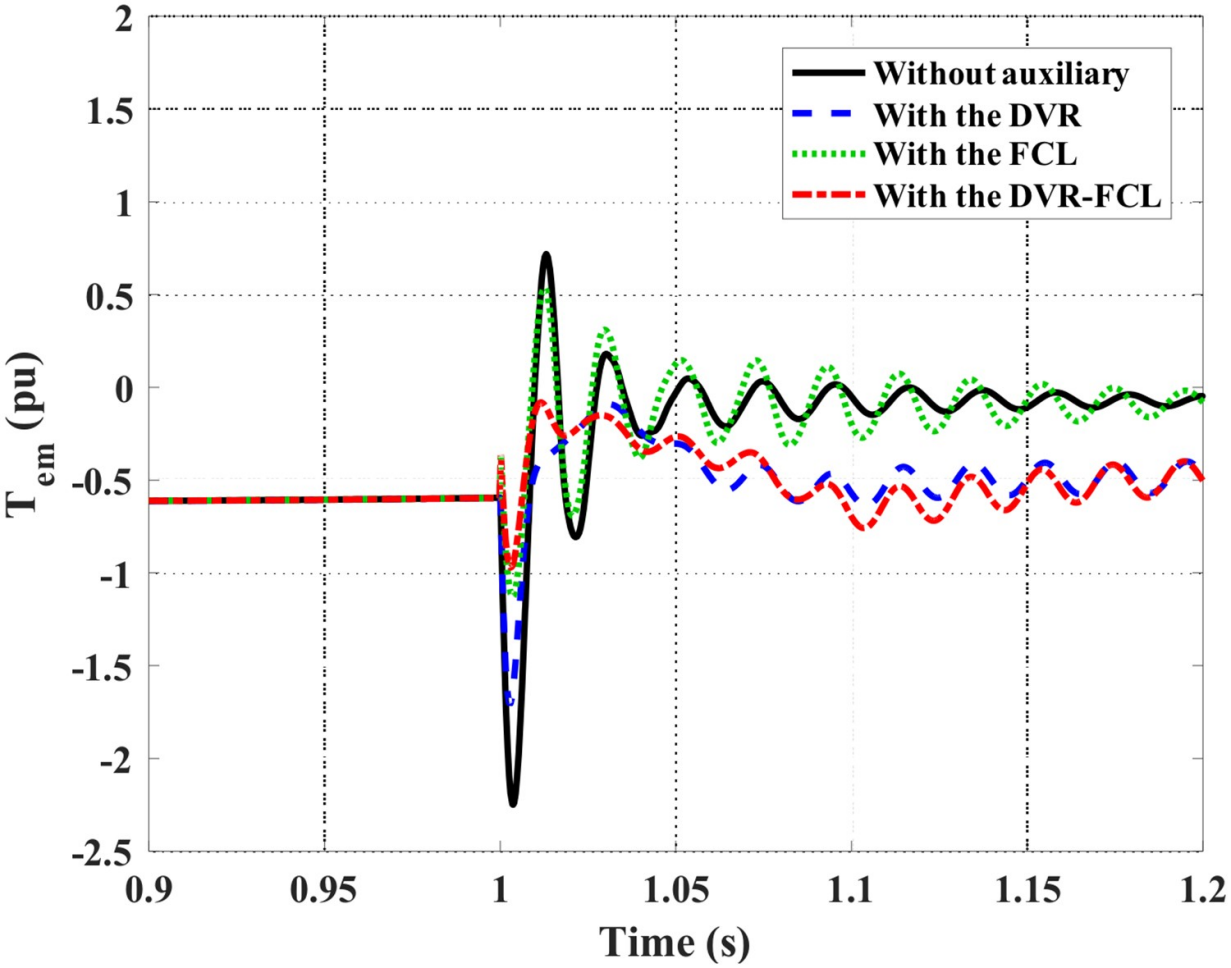
Electromagnetic torque of the DFIG subject to the symmetrical fault.

**Fig 12:**
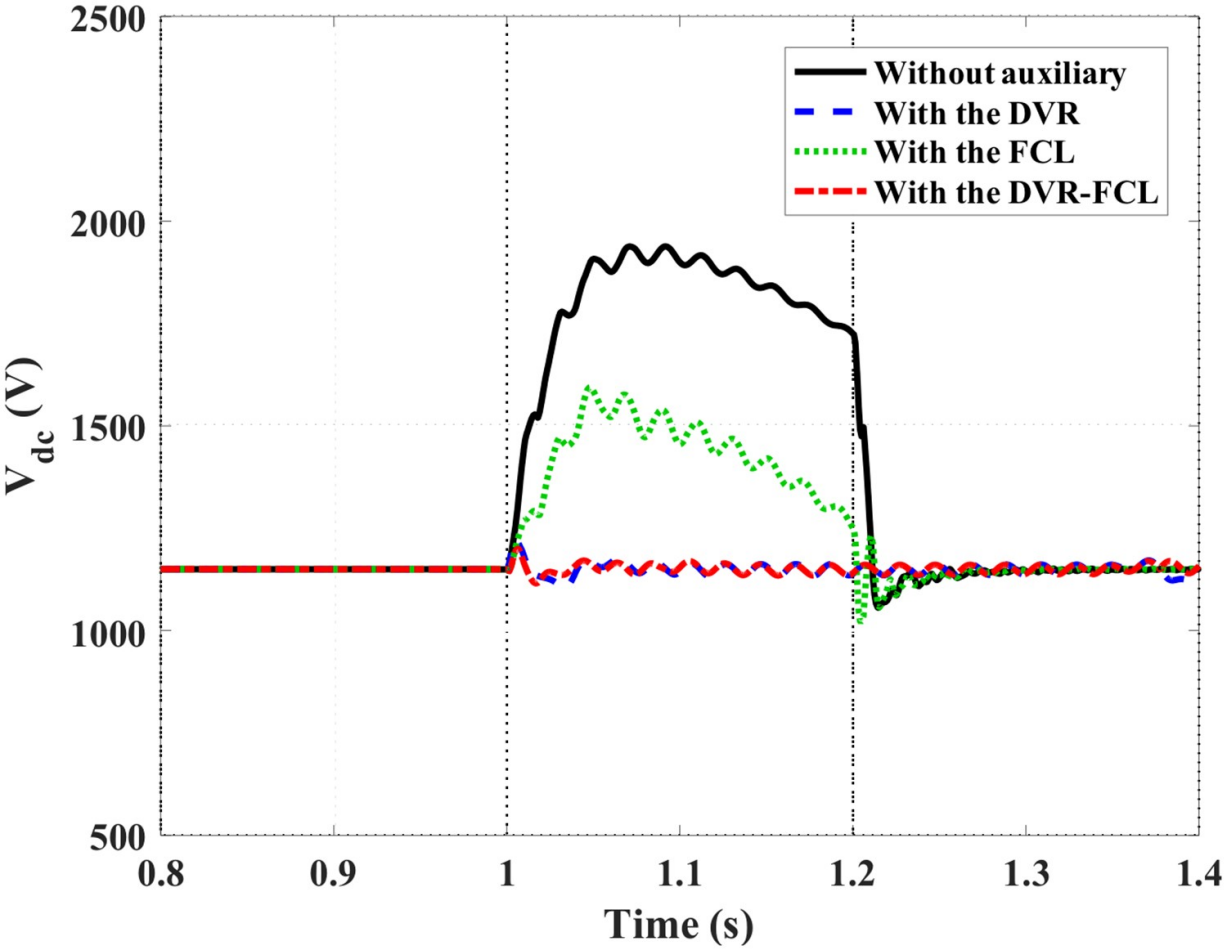
DC-link voltage of the DFIG subject to the symmetrical fault.

Figs 10–12 show the DFIG active power, electromagnetic torque and DC-link voltage during the fault, and the performance data of the selected four cases are indicated in Table 2. From the results, the combined DVR-FCL provides the best contributions in stabilizing the DFIG. The generator power is controlled at ~1 MW, and the electromagnetic torque is inhibited at 0.97 pu.

**Table 2:** Comparison of various methods on the DFIG subject to the symmetrical fault.

Items	Without auxiliary	With the DVR	With the FCL	With the DVR-FCL
Stator current	4.1 pu	2.1 pu	2.25 pu	1.49 pu
Rotor current	3.49 pu	2.31 pu	1.74 pu	1.4 pu
Terminal voltage (RMS)	0.05 pu	0.36 pu	0.06 pu	0.37 pu
Electromagnetic torque	2.25 pu	1.71 pu	1.13 pu	0.97 pu
DC-link voltage	1.94 kV	1.23 kV	1.59 kV	1.19 kV

Based on the simulations of the DC-link overvoltage, a technical discussion is as follows. The DC-link voltage is mainly determined by its excess power and the GSC’s adjusting ability. For without auxiliary, the larger stator current makes the GSC leave the linear control region, and the higher rotor current causes a considerable oscillation power. It is inevitable that the DC-link voltage fluctuates and increases sharply. For with the FCL, the constrained rotor current makes the oscillation power be lowered, and due to the coupling between the rotor and stator, the GSC’s adjusting ability can be appropriately improved. As affected by these two contributions, the FCL reduces the overvoltage level, but keeps the voltage pattern as the case without auxiliary.

For that the DVR is adopted, the above two benefits can be mildly remained, and also an enhanced terminal voltage increases the power stability of the DFIG. The excess power at the DC-link is accordingly mitigated, and it is a significant difference from the case with the FCL. In consequence, the DVR well outperforms the FCL to stabilize the DC-link overvoltage. That is why the cases with the DVR and the combined DVR-FCL do not follow the same pattern as the case with the FCL.

### 5.2 Study of the asymmetrical fault

It is simulated that a double-phase (phase-A and phase-B) fault happens at *t* = 1 s, and the fault duration is 200 ms. For the reasons why the most common single-phase fault is not chosen, our research group is to assess the effectiveness of the proposed approach to handle an asymmetrical fault with higher severity. If the combined DVR-FCL is capable of solving the double-phase fault problem, it is believed that the approach is also competent for handling the single-phase fault.

**Fig 13:**
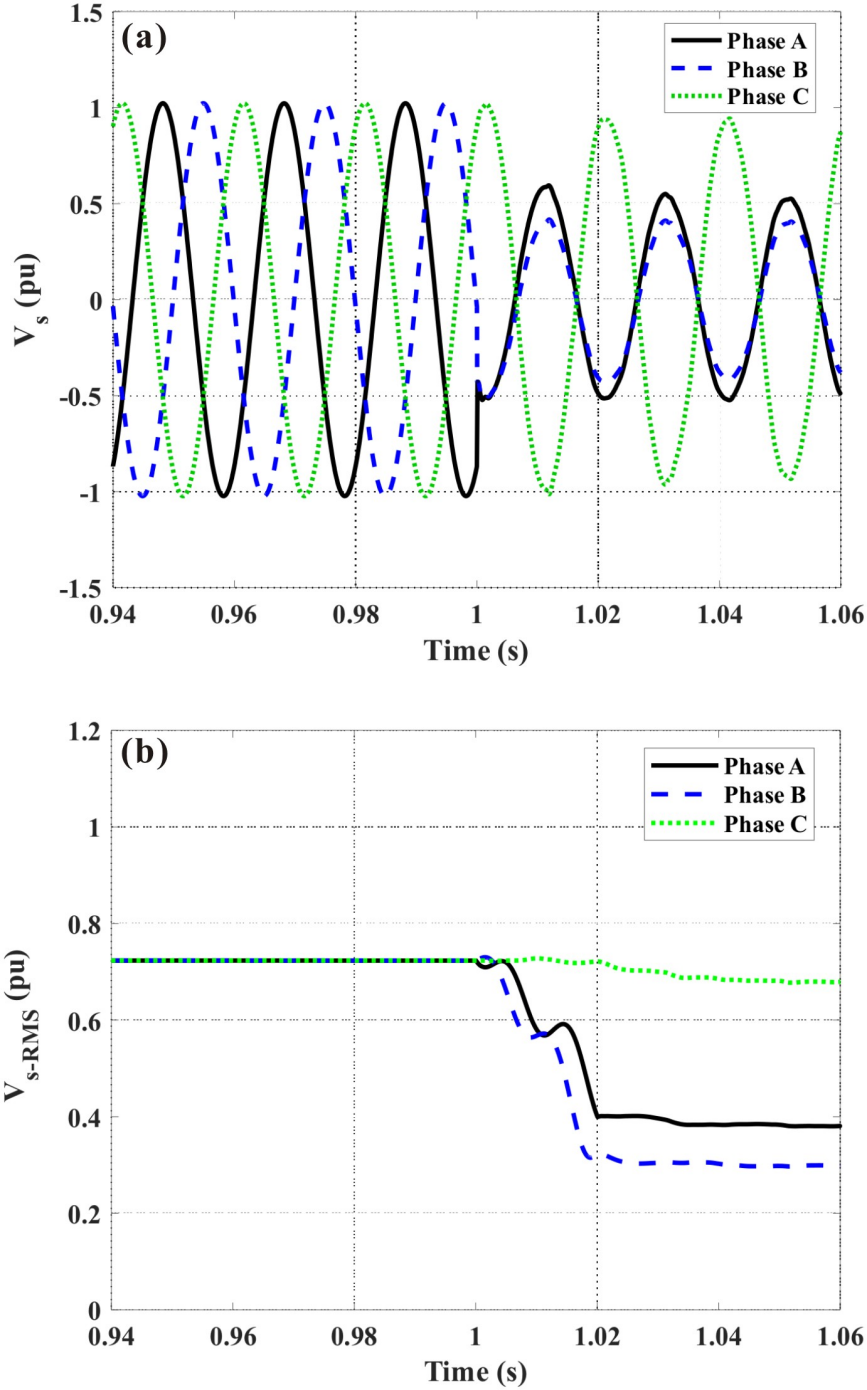
DFIG terminal voltage subject to the asymmetrical fault. (a) Instantaneous value, (b) RMS value.

**Fig 14:**
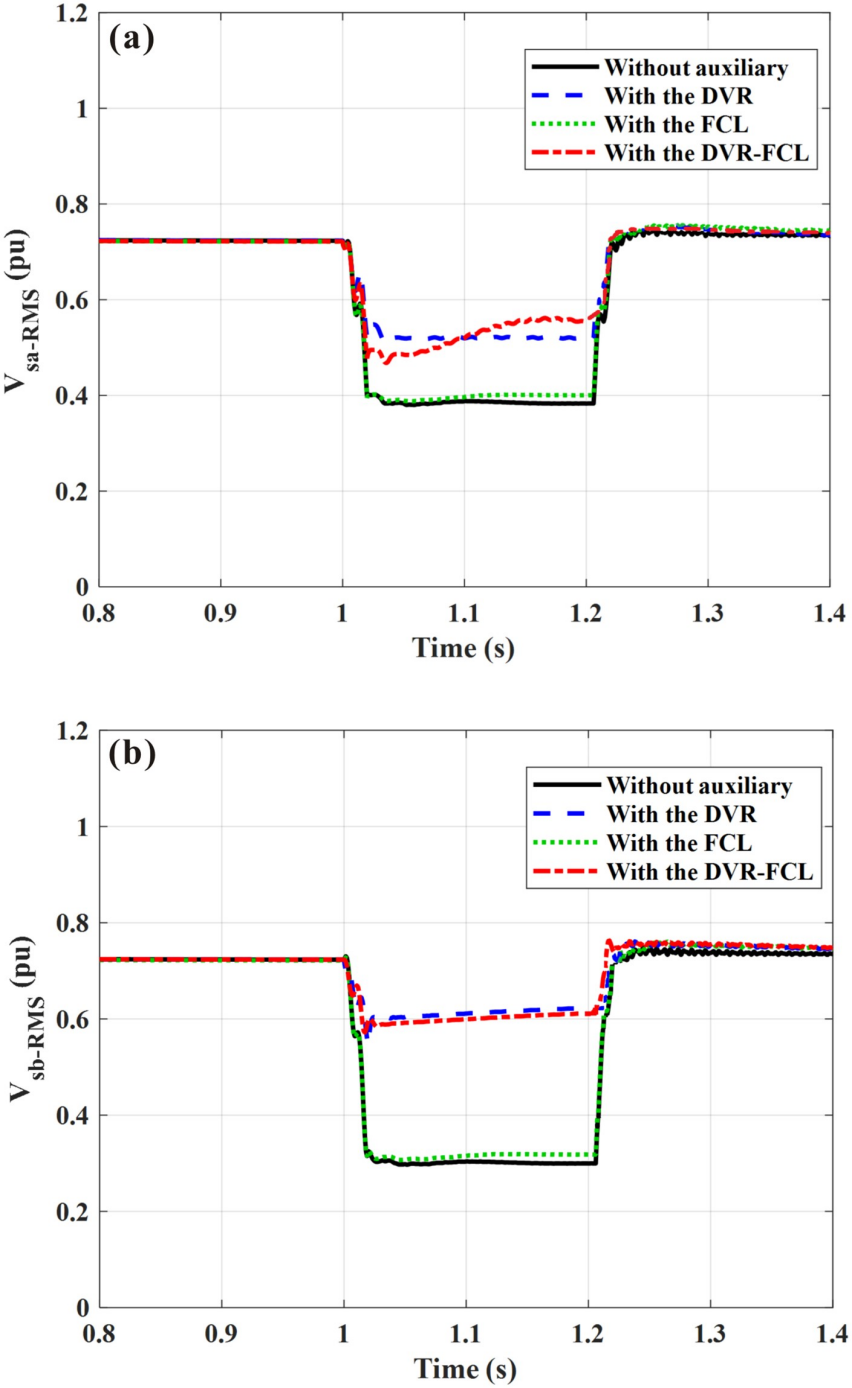
RMS value of the DFIG terminal voltage subject to the asymmetrical fault. (a) phase-A voltage, (b) phase-B voltage.

Fig 13 shows the DFIG terminal voltage during the asymmetrical fault. For without auxiliary, the RMS voltages of the two faulty phases (phase-A and phase-B) are decreased to 0.39 pu (55.2% of the nominal level) and 0.28 pu (39.6% of the nominal level), respectively. There is an ignorable voltage decline on the unfaulty phase, and the RMS voltage is kept at 94% of the nominal level. Fig 14 indicates the voltage compensation effects by various methods. It is clearly seen that the combined DVR-FCL brings the best voltage improvement. The DVR will firstly inject the voltage compensation on the two faulty phases, whose voltages are adjusted to be approximately equal with each other. Later, the inductive FCL will be activated to insert the desirable current-limiting inductance to the rotor circuit. From the results, the RMS voltages of the two faulty phases are enhanced to 0.56 pu (79.2% of the nominal level) and 0.61 pu (86.3% of the nominal level) by using the combined DVR-FCL, and the voltage drop rates are about 20.8% and 13.7%, respectively.

**Fig 15:**
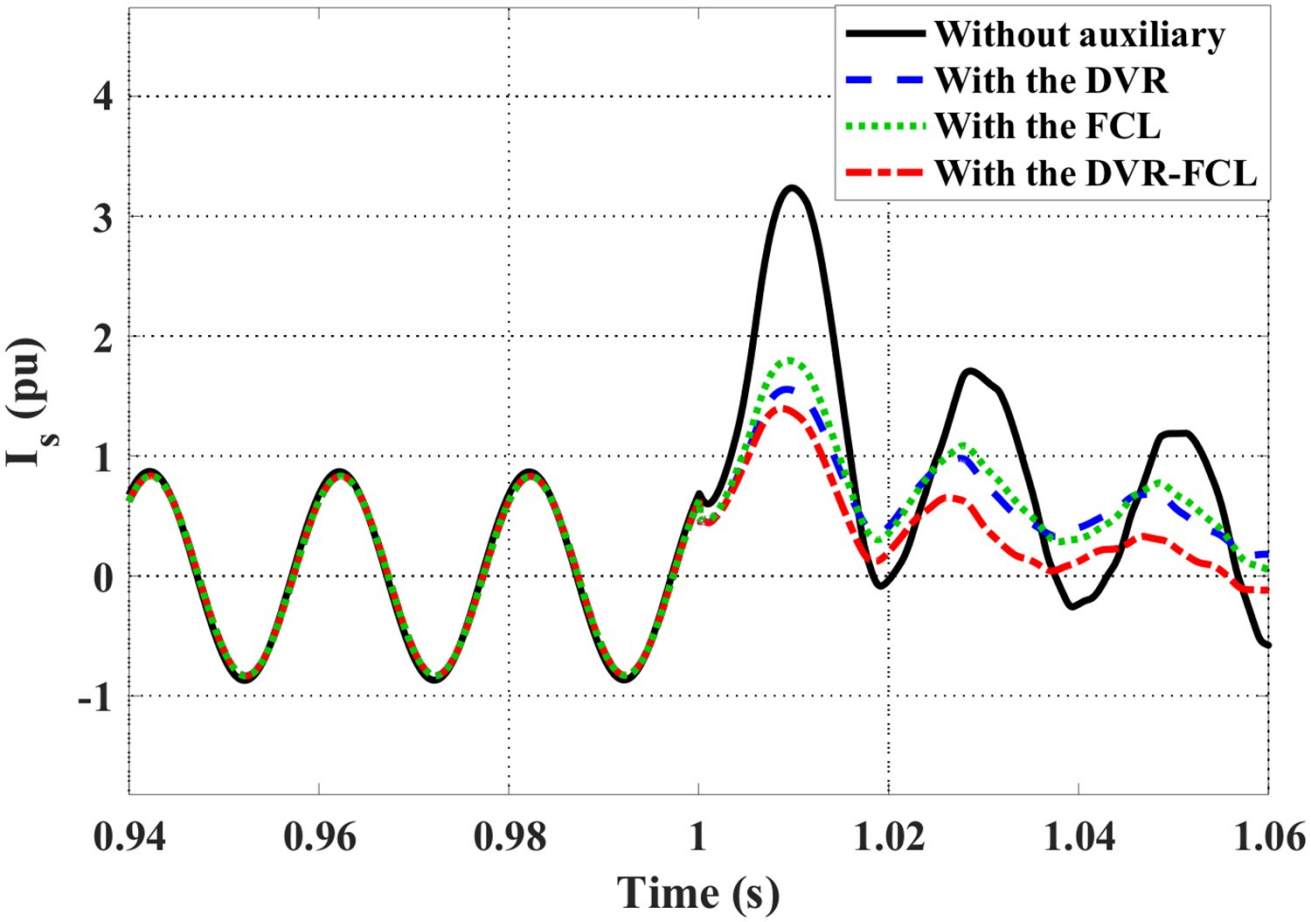
DFIG stator current subject to the asymmetrical fault.

**Fig 16:**
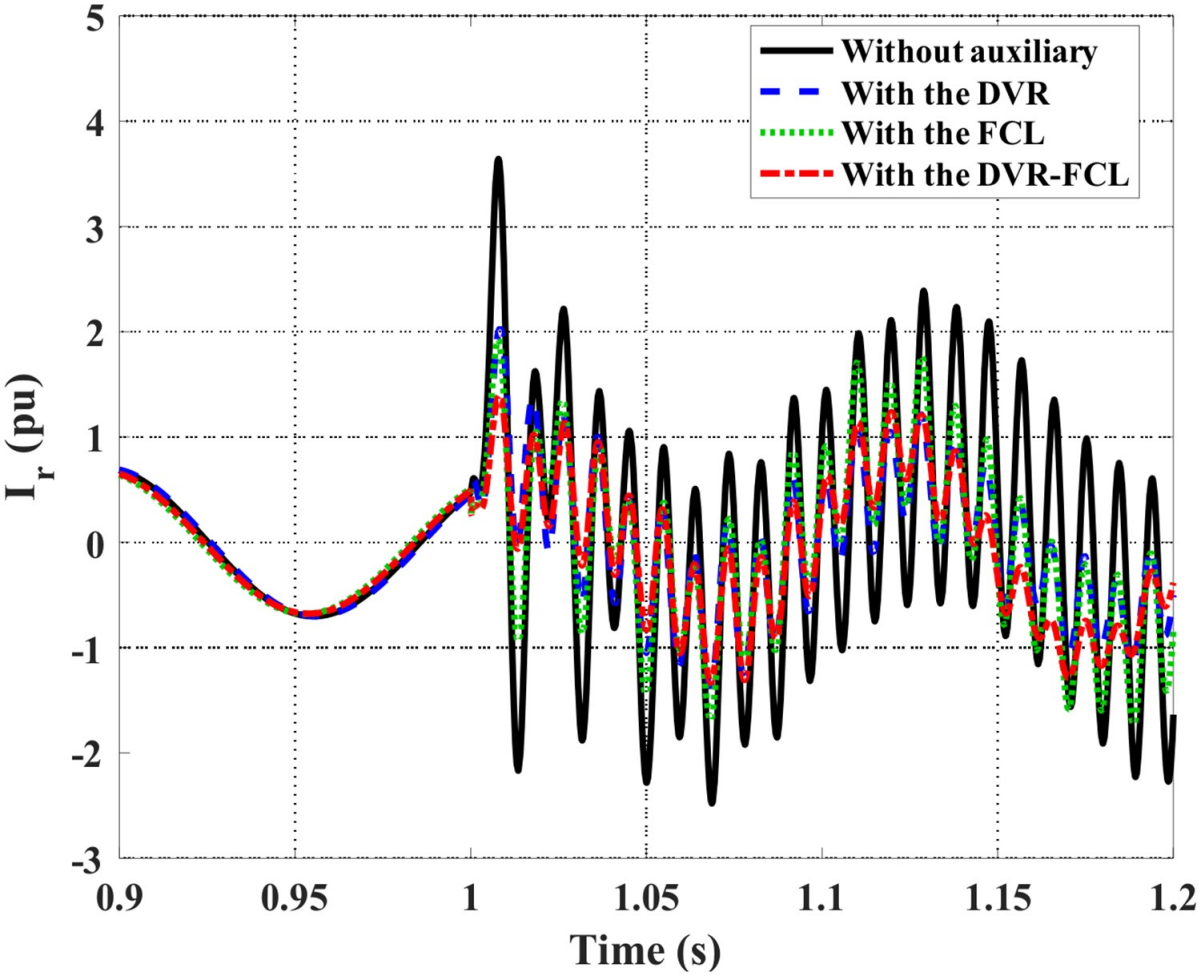
DFIG rotor current subject to the asymmetrical fault.

**Fig 17:**
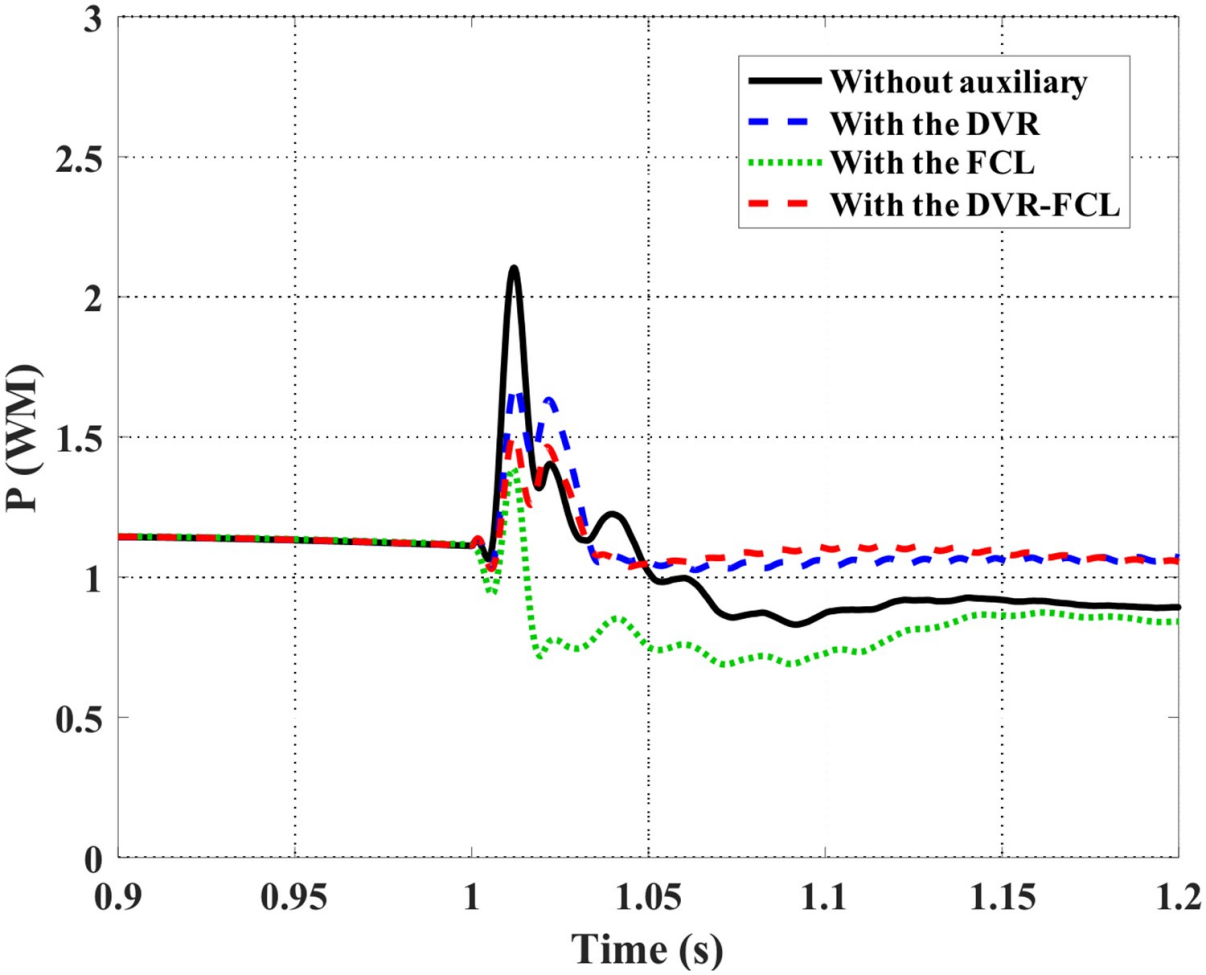
Active power of the DFIG subject to the asymmetrical fault.

**Fig 18:**
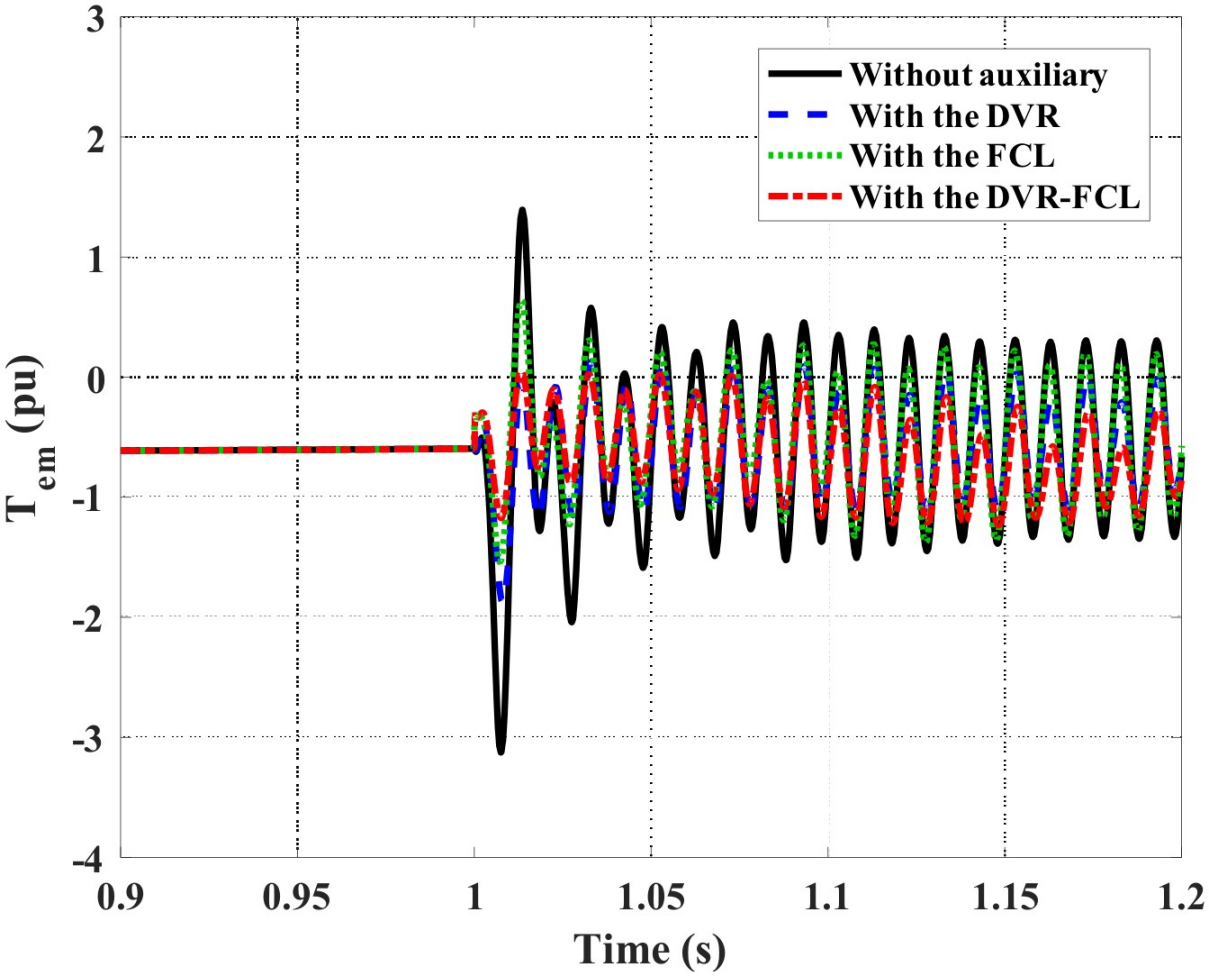
Electromagnetic torque of the DFIG subject to the asymmetrical fault.

**Fig 19:**
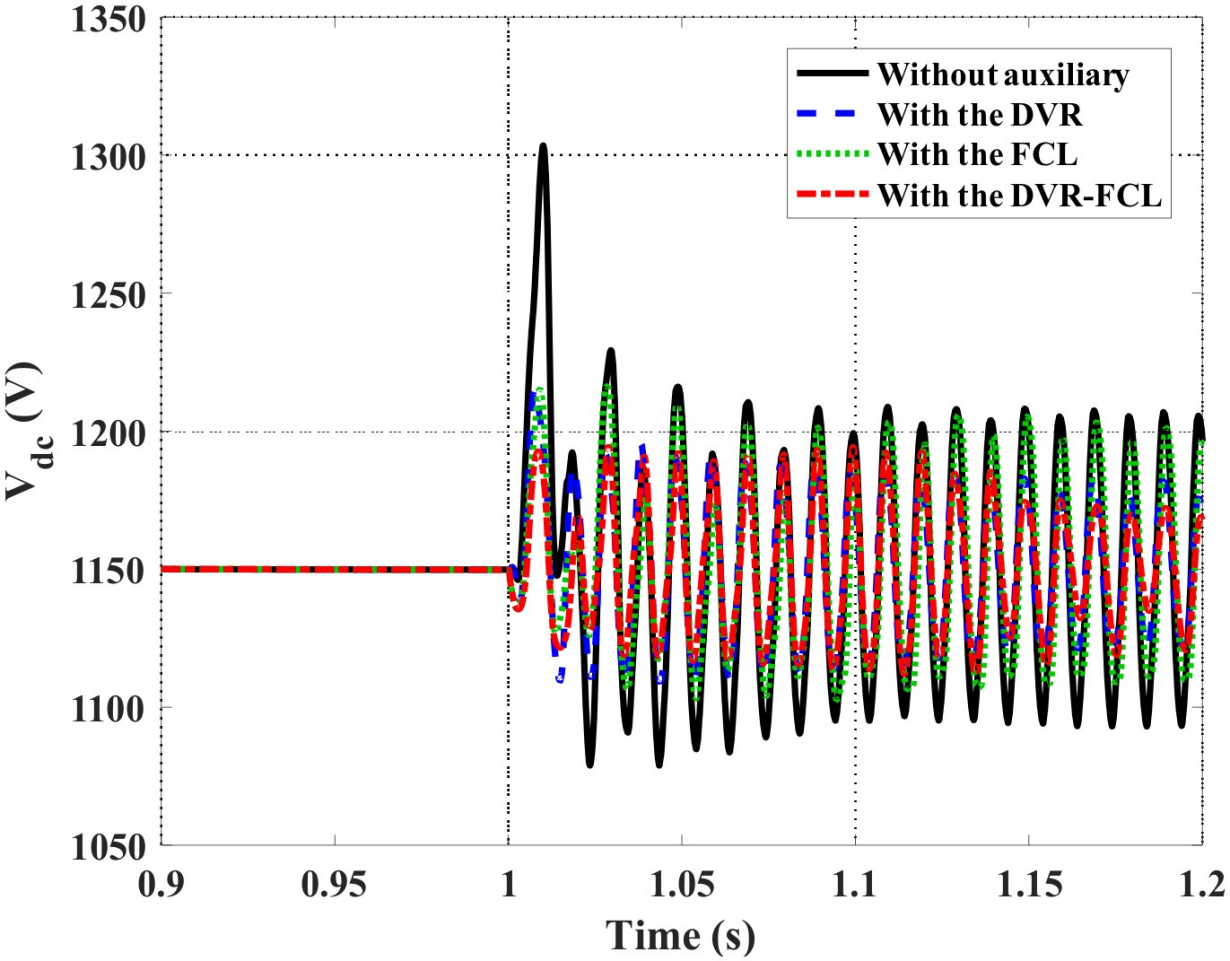
DC-link voltage of the DFIG subject to the asymmetrical fault.

Figs 15–19 show the DFIG stator and rotor currents, active power, electromagnetic torque and DC-link voltage subject to the asymmetrical fault. The detailed comparison of various methods is listed in Table 3. Herein, the single action of the DVR or the FCL may not avoid disconnection of the DFIG, since the rotor current is exactly around its safety limit. After the combined DVR-FCL is utilized, the rotor current is suppressed to 1.35 pu, and the current-limiting ratio is up to 63%.

**Table 3:** Comparison of various methods on the DFIG subject to the asymmetrical fault.

Items	Without auxiliary	With the DVR	With the FCL	With the DVR-FCL
Stator current	3.28 pu	1.54 pu	1.81 pu	1.39 pu
Rotor current	3.64 pu	2.03 pu	1.92 pu	1.35 pu
Terminal voltage (RMS)	0.38 pu	0.54 pu	0.39 pu	0.56 pu
Electromagnetic torque	3.12 pu	1.88 pu	1.55 pu	1.17 pu
DC-link voltage	1.31 kV	1.21 kV	1.22 kV	1.19 kV

The maximum power fluctuation is ~1 MW when without auxiliary. For that the combined DVR-FCL is used, the GSC and RSC both operate in the linear regions, and multiple benefits are obtained for power stabilization of the DFIG. One is to limit the maximum fluctuation within the level of 0.4 MW, and the other is to keep the generator power at the level of 1.08 MW.

As compared to the symmetrical fault, it is clearly seen that the asymmetrical fault causes more obvious oscillations in the electromagnetic torque and the DC-link voltage. With regard to the contributions of the combined DVR-FCL, the maximum electromagnetic torque is reduced from 3.12 pu to 1.17 pu, and it is helpful to alleviate mechanical stress on the turbine. Furthermore, the DC-link voltage is limited to 1.19 kV, and the oscillation range is controlled within 60 V.

## 6 Conclusions

This paper proposes an efficient LVRT scheme based on the coordination control of a DVR and an inductive FCL for a DFIG. Theoretical investigation and simulation analysis are done to validate the effectiveness of the proposed scheme. The combined DVR-FCL can powerfully decrease the fault currents in the DFIG stator and rotor, and perform visible voltage stabilization on the generator terminal and the DC-link. Additionally, the combined DVR-FCL enables to well strengthen the DFIG power stability and suppress the electromagnetic torque within the safety limit. In consequence, the risks to cause damage of the converters are avoided, and an adequate LVRT operation is realized for the DFIG under symmetrical and asymmetrical faults.

In the near future, the follow-on tasks for the proposed approach will be carried out, and they include parameter optimization, economic evaluation and prototyping test of the combined DVR-FCL in the DFIG. The specific research schemes and results will be addressed in other reports.

## Abbreviations

0: Initial time
*cs*: Controllable switch
ct1: Primary coil of coupling transformer
ct2: Secondary coil of coupling transformer
DC: Direct current
DFIG: Doubly fed induction generator
DVR: Dynamic voltage restorer
*e*: Electromagnetic force [V]
*f*: Fault
FCL: Fault current limiter
GSC: Generator side converter
*i*: Current [A]
*k*: Coupling factor [-]
*L*: Inductance [H]
LVRT: Low-voltage ride-through
*m*: Mutual
*Max*: Maximum
MPPT: Maximum power point tracking
*R*: Resistance [Ω]
*r*: Rotor
RE: Renewable energy
*ref*: Reference
RMS: Root-mean-square
RSC: Rotor side converter
*s*: Stator
SMES: Superconducting magnetic energy storage
*sys*: System
*V*: Voltage [V]
WT: Wind turbine
*X*: Reactance [Ω]
*Z*: Impedance [Ω]
*σ*: Leakage
*Ψ*: Flux [T]
*ω*: Angular velocity [rad/s]
